# US Medicaid program: An analysis of the spending and utilization patterns for antidepressants from 2017 to 2021

**DOI:** 10.1016/j.rcsop.2023.100392

**Published:** 2023-12-05

**Authors:** Mohamed Elmarasi, Brian Fuehrlein

**Affiliations:** a Department of psychiatry, Nassau University Medical Center, United States; b Psychiatry, Yale University, New Haven, CT, United States

**Keywords:** US Medicaid, Antidepressants, Spending patterns, Utilization patterns, Medication expenditure

## Abstract

**Background:**

Major depressive disorder (MDD) is a serious mental health condition that contributes to health complications, financial burden and death. In 2020, about one in five US adults had a lifetime diagnosis of MDD**.** With Major Depressive Disorder (MDD) being a common mental health concern, it is important to understand treatment patterns within public health systems like Medicaid, as they play a crucial role in providing care to diverse populations.

**Objective:**

The study investigated antidepressant usage and market distribution in the Medicaid Program. By doing so, the study aimed to provide insights into how these trends reflect broader changes in mental health treatment practices and policy implications within the Medicaid system during the study period.

**Methods:**

Public Medicaid data from 2017 to 2021 were analyzed, focusing on 30 FDA-approved antidepressants. Spending and prescription data were aggregated using Excel and Python.

**Results:**

The total US Medicaid expenditure on antidepressants increased from about $1 billion dollars in 2017 to $1.12 billion dollars in 2021, an increase of about 10%. Consistently, SSRIs were the class of antidepressants that Medicaid spent the most on. The highest Medicaid spending on a single antidepressant in 2017 and 2018 was bupropion. During the remaining years of the study (2019, 2020, 2021) Medicaid appropriated most funds toward Vortioxetine. The total number of antidepressant prescriptions increased from 52 million scripts to 59 million scripts (an increase of about 14%).

**Conclusions:**

The increase in Medicaid spending on antidepressants during the study period can be explained by an increase in utilization (a 14% increase in antidepressant prescriptions from 2017 to 2021), and a shift toward prescribing newer more costly antidepressants (like SSRIs and others) and away from prescribing older, less costly antidepressants like monoamine oxidase inhibitors (MAOIs) and tricyclic antidepressants (TCAs).”

## 1 Introduction

Major depressive disorder (MDD) is a serious mental health condition that contributes to health complications, financial burden and death.^1^^,^^2^ In 2020, about one in five US adults had a lifetime diagnosis of MDD.^1^ The diagnosis is based on the DSM-5 criteria, which requires symptoms to persist for at least two weeks. MDD significantly impacts quality of life and functional ability. In 2020, the overall economic burden of MDD was estimated to be $32.2 billion dollars, with the largest portion of the amount due to workplace costs.

Antidepressants are a first-line treatment for MDD. They are classified into categories based on their mechanism of action (see Table 1 for details). The oldest classes of antidepressants are MAOIs and TCAs; the development of both began in the 1950s. MAOIs and TCAs have largely been replaced by newer classes of medications which have better safety profiles and less severe side effects.^3^ The advent of SSRIs presented a major advancement in the treatment of depression. Fluoxetine was the first SSRI to obtain the FDA approval in 1987. Since then, citalopram, escitalopram, paroxetine, sertraline, vilazodone, and most recently in 2013, vortioxetine have obtained approval. Despite the significance of SSRIs in the treatment of depression, they have many shortcomings including delayed onset of action, partial response or no-response, and side effects. To address some of the shortcomings of the SSRIs, SNRIs were developed. These include venlafaxine (the first one obtaining approval in 1993), duloxetine, desvenlafaxine and levomilnacipran.^4^ Bupropion and mirtazapine are atypical antidepressants that were FDA-approved in 1985 and 1996, respectively. More recently, in 2019, the FDA approved two rapid-acting antidepressants, esketamine and brexanolone.

**Table 1:** FDA approved antidepressants (included in our study).

Category	Generic Name	Brand Name
Selective Serotonin Reuptake Inhibitors (SSRIs)	Citalopram	Celexa
	Escitalopram	Lexapro
	Paroxetine	Paxil, Paxil CR, Pexeva
	Fluoxetine	Prozac, Prozac Weekly
	Vortioxetine	Trintellix
	Vilazodone	Viibryd
	Sertraline	Zoloft
Serotonin-Norepinephrine Reuptake Inhibitors	Duloxetine	Cymbalta
(SNRIs)	Venlafaxine	Effexor, Effexor XR
	Levomilnacipran	Fetzima
	Desvenlafaxine	Pristiq, Khedezla
Tricyclic and Tetracyclic Antidepressants	Amoxapine	Asendin
	Amitriptyline	Elavil
	Maprotiline	Ludiomil
	Desipramine	Norpramin
	Nortriptyline	Pamelor
	Doxepin	Sinequan
	Trimipramine	Surmontil
	Imipramine	Tofranil
	Protriptyline	Vivacti
Atypical Antidepressants	Trazodone	Desyrel
	Nefazodone	Serzone
	Mirtazapine	Remeron
	Bupropion	Wellbutrin, Wellbutrin SR, Wellbutrin XL
Monoamine Oxidase Inhibitors (MAOIs)	Selegiline	Emsam (Skin Patch)
	Isocarboxzaid	Marplan
	Phenelzine	Nardil
	Tranylcypromine	Parnate
*N*-methyl d-aspartate (NMDA) Antagonist	Esketamine	Spravato (nasal spray)
Gamma-aminobutyric acid (GABA) receptor positive modulators	Brexanolone	Zulresso

In the US, the largest single payer for mental health care is Medicaid.^5^ Furthermore, studies have found that Medicaid enrollees have a higher prevalence and severity of MDD than privately insured individuals.^6^ The prevalence of MDD in the Medicaid population was estimated to be 18% to 20%.^4^ In 1991, the total Medicaid spending on SSRIs and SNRIs was $64.5 million. It increased significantly to $2 billion in 2004 and then decreased to $755 million in 2018.^7^

This study aimed to examine the usage patterns of antidepressants and the portion of the market controlled by different medications in Medicaid spending. This clarity may inform clinicians and policymakers about a key segment of the antidepressant market. Additionally, we hope to show the impact of newer antidepressants in the market. Finally, we hope to elucidate the competition between antidepressants manufacturing companies, which may be of interest to policy makers.

## 2 Methods

Publicly available, de-identified Medicaid drug spending and utilization data from 2017 to 2021 were used in the study. The dataset was accessed from the Centers for Medicare & Medicaid Services (CMS) at https://data.cms.gov/summary-statistics-on-use-and-payments/medicare-medicaid-spending-by-drug/medicaid-spending-by-drug/data. Data represent national spending by state agencies for outpatient drugs. The total Medicaid spending on any drug is inclusive of both state and Federal reimbursements in addition to any other fees. However, it is not affected by Medicaid rebates. The Medicaid drug rebate program is an agreement between CMS and the drug manufacturers under which the drug manufacturers provide back part of the cost to the state. Therefore, the Medicaid drug rebate program drives down overall Medicaid spending on drugs. This makes the total spending an imperfect measure of total cost, which should be noted while interpreting results of the study.

Thirty FDA-approved antidepressants were identified. They were divided into subclasses as listed in Table 1. The data were aggregated to calculate overall spending and total prescriptions for each of the antidepressants and each subclass of antidepressants for all years of the study. Additionally, market share was calculated as the proportion of total payment or total prescriptions. All analyses were conducted using Excel and Python software.

## 3 Results

**Fig. 1:**
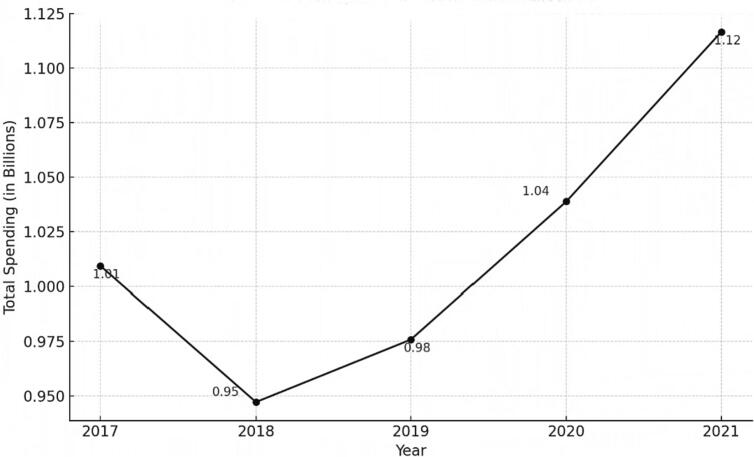
Total Medicaid spending on antidepressants from 2017 to 2021.

**Fig. 2:**
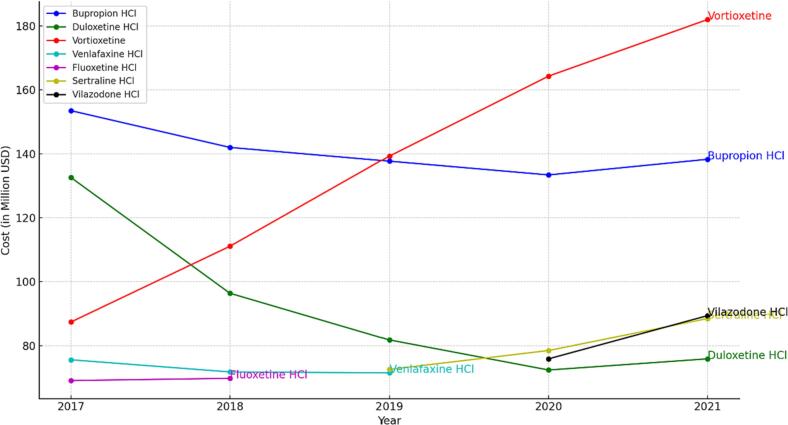
Annual expenditure on individual antidepressant medications within the Medicaid program.

Total US Medicaid expenditures for antidepressants changed from about $1 billion dollars in 2017 to $1.12 billion dollars in 2021, an increase of about 10% (Fig. 1). Consistently throughout our study, SSRIs represented the largest expenditure. For example, in 2017, Medicaid paid over $363 million dollars for SSRIs alone, comprising about 39.9% of total Medicaid spending on antidepressants that year. And in 2021, Medicaid paid over $540 million dollars on SSRIs, comprising about 48% of its total spending on antidepressants. The highest Medicaid spending on a single antidepressant in 2017 and 2018 was on bupropion. In 2018, Medicaid spent $142 million dollars on bupropion prescriptions. During the remaining years under study (2019, 2020, 2021) Medicaid spent the most on Vortioxetine. In 2021, Medicaid spent over $181 million USD on Vortioxetine prescriptions (Fig. 2).

Total number of antidepressant prescriptions increased from 52 million scripts in 2017 to 59 million scripts (an increase of about 14%). Throughout the study years (2017–2021), SSRIs were the most prescribed comprising about 49% of total prescriptions.

**Fig. 3:**
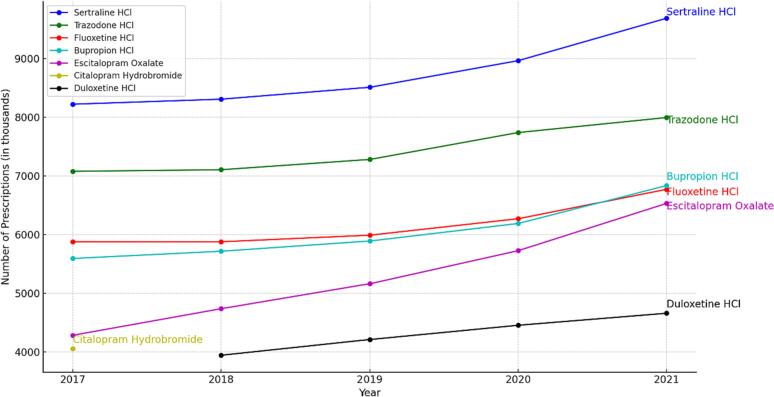
Most prescribed antidepressants throughout the study years.

The most prescribed antidepressants were sertraline, trazodone, and fluoxetine respectively. This order was stable from 2017 to 2020. However, in 2021, bupropion became the third most prescribed antidepressant instead of fluoxetine (Fig. 3). In 2021, sertraline had 9.6 million prescriptions followed by trazodone with 7.9 million prescriptions and bupropion had 6.8 million prescriptions. The most prescribed SNRI was Duloxetine with 4.6 million prescriptions in 2021. Amitriptyline was the most prescribed TCA with 2.4 million prescriptions in 2021. Esketamine prescriptions in 2019 were only 2.7 thousand (it was FDA-approved for treatment resistant depression on March 5, 2019). However, by 2021 the esketamine prescriptions increased to 22.9 thousand prescriptions (an increase of 156.85%). Additionally, another recently approved antidepressant, brexanolone, which was FDA approved (on March,19, 2019) for the treatment of postpartum depression in adults had 46 prescriptions in 2020 and 24 prescriptions in 2021.

## 4 Discussion

Our results showed that the increase in Medicaid spending on antidepressants during the study period can be explained by an increase in utilization (a 14% increase in antidepressant prescriptions from 2017 to 2021), and a shift toward prescribing newer more costly antidepressants (like certain SSRIs) and away from prescribing older, less costly antidepressants.

### 4.1 Trends in utilization

During the study years, there was a shift toward SSRIs and away from MAOIs and TCAs. For example, SSRIs had about 25 million prescriptions in 2017, and this number increased to 28.9 million prescriptions in 2021. Additionally, SNRIs had 7.4 million prescriptions in 2017 and this number increased to 8.8 million in 2021. Meanwhile, total number of TCAs prescriptions fell from 4.2 million prescriptions in 2017 to 3.9 million prescriptions in 2021. Similarly, total number of MAOI prescriptions fell steadily from 7620 in 2017 to 7010 in 2021.

The most prescribed SSRIs were sertraline and fluoxetine, respectively. The number of sertraline prescriptions has grown consistently from 8.2 million prescriptions in 2017 to 9.6 million prescriptions in 2021. During all study years, sertraline was the most prescribed antidepressant. Preference for sertraline over other antidepressants may be due to its wide dose range (25–200 mg/day), favorable side-effects and efficacy profiles, low risk for drug interactions, and its FDA-approval to treat many conditions.^8^ The most prescribed SNRI was duloxetine with over 4.6 million prescriptions in 2021. Duloxetine is the only antidepressant that is FDA-approved to treat neuropathic pain. Trazodone was the most prescribed atypical antidepressant (and the number-two most prescribed antidepressant during all study years) with over 7.9 million prescriptions in 2021. This relatively large number may be because trazodone is one of the most prescribed sleep aids in the US. Although it is FDA-approved only for the treatment of depression, its off-label use as a sleep aid surpassed its use for depression.^9^

Among TCAs, the most prescribed was amitriptyline. However, amitriptyline prescriptions were consistently decreasing, from 2.7 million prescriptions in 2017 to 2.4 million prescriptions in 2021. The most prescribed MAOI varied between selegiline, phenelzine, and tranylcypromine with phenelzine being the most prescribed MAOI in 2017 and tranylcypromine being the most prescribed in 2021. However, total number of MAOI prescriptions fell steadily from 7620 in 2017 to 7010 in 2021. As we alluded to before, TCAs and MAOIs were the two first classes of antidepressants to be discovered. Their advent paved the way to the discovery of newer antidepressants. However, newer antidepressants have better safety profiles, are more heavily marketed, and relatively easy to administer. These factors led to a decrease in prescriptions for MAOIs and TCAs.^10^

### 4.2 Trends in spending

The highest Medicaid spending on a single antidepressant in 2017 and 2018 was on bupropion despite it being the fourth most prescribed antidepressant. From 2019 to 2021, the largest expenditure was on Vortioxetine. For example, in 2021, Medicaid spent about 16% of its total antidepressant budget—approximately $181 million—on Vortioxetine. Also in 2021, by contrast, Medicaid spent about $88 million on Sertraline, despite it being the most prescribed antidepressant. The discrepancy between utilization rate and payment proportion is likely because sertraline is available in generic form, which drove the average spending per prescription down to only about $30 in 2021. On the other hand, vortioxetine is only available under the brand name Trintellix with an average spending per prescription of $444 in 2021. Vortioxetine is a multimodal antidepressant that enhances serotonin release; it is one of the most expensive antidepressants.^11^^,^^12^ It is the first antidepressant to demonstrate benefits in both cognitive symptoms and depression.^13^,^14^ Whether this benefit of vortioxetine justifies its elevated cost in all patients is an area that needs further studying.

The cost of name-brand medications has risen over time. Per-prescription cost of Zoloft (sertraline) increased from $382 in 2017 to $578 in 2021 (a 51% increase), Cymbalta (duloxetine) from $271 in 2017 to $396 in 2021 (a 46% increase), and Trintellix (vortioxetine) from $353 in 2017 to $444 in 2021 (an increase of 25%). Conversely, cost of generic medications decreased from an average cost per prescription in 2017 of $53 to $47 in 2021 (an 11% decrease).

### 4.3 Cost containment

Medicaid implemented a variety of cost-containment strategies. These include co-payments (requiring patients to contribute toward the cost of their prescriptions), generic mandates (mandating use of the generic form when available before considering brand name), dispensing limits (having a cap on the number of days before a refill is needed), and prior authorization (obtaining Medicaid approval before dispensing prescriptions).^15^ While it's important to contain the cost of antidepressants, it's equally important not to sacrifice accessibility of medications or continuity of services for individuals in need of mental health services. Evidence suggests that aggressive cost-containment strategies among vulnerable patients can negatively affect adherence with medications.^16^ Therefore, policymakers should weigh the risks and benefits of cost containment especially among more vulnerable populations.

### 4.4 Limitations

The Medicaid Drug Spending Dashboard datasets utilized for this analysis did not include sociodemographic details such as age, gender, and race, number of beneficiaries who were given antidepressants, and diagnostic codes. Furthermore, it's important to note that reported costs did not consider possible rebates and discounts offered to states. Despite these limitations, our data were adequate for estimating overall Medicaid spending on antidepressants and its trends. Findings of this study should be understood considering the study's limitations.

## 5 Conclusion

Our study revealed a significant shift in Medicaid's antidepressant prescribing patterns from 2017 to 2021, favoring newer SSRIs and SNRIs like sertraline and duloxetine over older TCAs and MAOIs. This trend reflected an evolving understanding of the efficacy and safety profiles of these drugs, as well as changes in pharmaceutical marketing and administration. While sertraline remained the most prescribed antidepressant, there had been a noticeable increase in prescriptions for costlier, brand-name medications like Trintellix (vortioxetine). This shift led to a rise in Medicaid spending on antidepressants, with newer drugs commanding higher prices despite their uncertain cost-benefit balance in all patient populations. The study's limitations, including the absence of detailed sociodemographic data and the exclusion of potential rebates in cost analysis, pointed to areas for further research. Ultimately, this study emphasized the importance of ongoing monitoring and a careful approach to cost containment in Medicaid's antidepressant prescriptions, ensuring both financial responsibility and accessibility of antidepressants.

## CRediT authorship contribution statement

**Mohamed Elmarasi:** Writing – review & editing, Writing – original draft, Software, Methodology, Formal analysis, Conceptualization. **Brian Fuehrlein:** Validation, Supervision, Conceptualization.

## Declaration of Competing Interest

The authors declare that they have no conflicts of interest related to this study.
